# Fabrication of Polymeric Antireflection Film Manufactured by Anodic Aluminum Oxide Template on Dye-Sensitized Solar Cells

**DOI:** 10.3390/ma10030296

**Published:** 2017-03-15

**Authors:** Jenn-Kai Tsai, Yu-Shin Tu

**Affiliations:** Department of Electronic Engineering, National Formosa University, Yunlin 632, Taiwan; tuyushin993@gmail.com

**Keywords:** anodic aluminum oxide (AAO), subwavelength structure (SWS), antireflection, polymethyl methacrylate (PMMA), dye-sensitized solar cell (DSSC), conversion efficiency

## Abstract

In this study, high energy conversion efficient dye-sensitized solar cells (DSSCs) were successfully fabricated by attaching a double anti-reflection (AR) layer, which is composed of a subwavelength moth-eye structured polymethyl methacrylate (PMMA) film and a polydimethylsiloxane (PDMS) film. An efficiency of up to 6.79% was achieved. The moth-eye structured PMMA film was fabricated by using an anodic aluminum oxide (AAO) template which is simple, low-cost and scalable. The nano-pattern of the AAO template was precisely reproduced onto the PMMA film. The photoanode was composed of Titanium dioxide (TiO_2_) nanoparticles (NPs) with a diameter of 25 nm deposited on the fluorine-doped tin oxide (FTO) glass substrate and the sensitizer N3. The double AR layer was proved to effectively improve the short-circuit current density (JSC) and conversion efficiency from 14.77 to 15.79 mA/cm^2^ and from 6.26% to 6.79%, respectively.

## 1. Introduction

Minimizing optical reflection in solar cells to improve conversion efficiency is always a major challenge. The literature has shown that there is still more than 30% of incident sunlight lost, even with adopting antireflection (AR) film in solar devices [1,2,3]. For example, silicon nitride AR coating has been widely used in solar cells since it is easy to fabricate; however, its performance is not satisfying due to its narrow-band and narrow-angle properties [4]. Multilayer AR film [4,5] also have been developed in order to further reduce the loss. However, the process instability, high cost, and high-temperature procedure limit the applications of these AR films. Besides multilayer coating, surface texturization by alkaline etching technique is an alternative method that has been widely used in silicon-based solar cells [6]. The idea arises from the gradual variation in the refractive index between air and silicon [6] that reduces the loss of light from reflection. A similar texturing technique using acid solution has also been applied to multi-crystalline silicon-based solar cells. On the other hand, subwavelength moth-eye structures have been explored in literature for decades, and it has been demonstrated that this structure has a low reflection over a broad spectral range [7,8]. E-beam lithography, photolithography, and nano-sphere lithography techniques are available for fabricating moth-eye patterns [2]. However, these techniques are time consuming and expensive. Therefore, a cheap and efficient technique to fabricate moth-eye structured AR layers is needed for photovoltaic devices.

Grätzel et al. reported a dye-sensitized solar cell that has a photoelectric conversion efficiency of 7.1% by using a TiO_2_ nano-porous electrode combined with dyes of metal ruthenium organic complexes and an electrolyte containing I^−^/I^3–^ redox couple [9]. Increasing photoelectric conversion efficiency and stability to achieve high performance dye-sensitized solar cells are the primary goals in this field. Major factors that influence the conversion efficiency are the transparent conductive oxide (TCO), working electrode, sensitizer, electrolyte and counter electrode, etc., and optimization of these factors plays an important role. For example, the transparent conductive electrode must have at least 80% visible light transmittance, which limits the thickness of the electro-conductive film. However, a thick electro-conductive film is usually required in order to have sufficient conductivity.

In this paper, we propose a simple and cheap method to fabricate moth-eye structured additional AR layers that combine with dye-sensitized solar cell (DSSC) devices. The moth-eye structure was fabricated based on the replication technology of an anodic aluminum oxide (AAO) template. The additional AR layer was made by polymethyl methacrylate (PMMA) because of its high resolution, low cost and optical transparency in the wavelengths between visible and near-infrared regions. The additional PMMA AR films have gradual variation in the refractive index that can effectively absorb incident light and greatly reduce reflectivity [10]. Moreover, since the thin PMMA films are flexible, they can be mounted on curved optical structures for many interesting applications. Using PMMA or PDMS as anti-reflection layers on solar cells has been reported with great improvement on light harvest efficiency [10,11,12,13]. The efficiency increase can be up to 3%–5%. The process, however, needs heat treatment over 100 °C, which is not compatible for the process for fabricating DSSCs [14]. In this paper, we also developed a two-step process to overcome the issue.

## 2. Experimental

### 2.1. Fabrication of AAO Template

Super purity aluminum sheets (99.969%, Toyo Aluminium Foil, Osaka, Japan) of 0.25 mm thickness were used as a substrate to serve as the original mold. The AAO template was fabricated by using a two-step anodization process. In the first anodization process, the pre-treated aluminum sheets were immersed in 0.1 M oxalic acid dihydrate (SIGMA-ALDRICH 99.5%, diluted by DI water) at a constant voltage of 80 V and a temperature of 4 °C for 3 h. During the anodization, the solution was stirred by a pump circulation system. Then, the anodic alumina layer was removed by wet chemical etching in 6 wt % phosphoric acid (H_3_PO_4_) with 1.8 wt % chromic acid (H_2_CrO_4_) at 60 °C for 60 min. The last anodization process was processed under the same conditions as the first anodization process, but the anodization period was reduced to 75 s. After that, the pore diameter of the anodic alumina was further widened by immersing the sample in 6 wt % phosphoric acid at 40 °C for 12 min.

### 2.2. Spin-Coating Replication

350 kmol, 15 wt % PMMA solution dissolved in toluene was spin-coated on the AAO template at 1000 rpm rotation speed. The film then dried for 30 min at room temperature and baked at 200 °C in an oven for 30 min. The sample then was immersed in 6 wt % sodium hydroxide (NaOH) at 60 °C for 60 min to remove the AAO template. After that, the sample was washed with dilute hydrochloric acid (10%) and subsequently dried in an oven at 45 °C for 10 min. Finally, the subwavelength moth-eye structure PMMA film was obtained.

### 2.3. DSSC Fabrication

10 wt % TiO_2_ paste was prepared by mixing nanocrystalline TiO_2_ particles (TG–P25, Degussa, Shinjuku, Tokyo, Japan; the average nanoparticle diameter was about 25 to 30 nm) with tert-butyl alcohol and deionized water. The TiO_2_ paste was scraped by the doctor blading method on a transparent fluorine-doped-tin oxide (FTO) glass, which has sheet resistance 8 Ω/□. The sample then was compressed mechanically at a pressure of 279 kg/cm^2^. The post-heat treatment was done by two-step annealing, 150 °C for 90 min and 500 °C for 30 min in air, respectively, to form TiO_2_ photoanodes [15,16]. The 150 °C annealing temperature would decompose the residual organic compounds and the 500 °C annealing temperature would assist the interconnection of TiO_2_ nanoparticles (NPs), so that the loss of electron transmission lessens. The compressed and annealed TiO_2_ NP films were immersed in 0.3 mM N_3_ dye (cis-bis(dithiocyanato)-bis(4,4′-dicarboxylic acid-2,2′-bipyridine) ruthenium (II)) for 2 h. Subsequently, they were rinsed in acetonitrile for a few seconds to wash out unbound dyes and dried in an oven at 45 °C. The counter electrode was made by electroplating 1 nm-thick Pt thin film on indium-tin-oxide (ITO) glass. The FTO substrate with TiO_2_ NP photoanodes and dye molecules on it was then flipped and placed on the Pt counter electrode with a 50 μm-thick hot-melt polymer spacer. Sealing was accomplished by pressing the two electrodes together at 115 °C for a few seconds. The redox electrolyte, consisting of 0.5 M LiI, 0.05 M I_2_, 0.5 M 4-tert-butylpyridine (TBP), and 1 M 1-propy1-2,3-dimethylimidazolium (DMPII) mixed into 3-methoxypropionitrile (MPN), was injected into the cell by capillary forces through an injecting hole that was previously made in the counter electrode by a drilling machine [17]. Finally, the hole was covered and sealed with a piece of hot-melt polymer to prevent leakage of the fluid-type electrolyte. The resulting active electrode area was approximately 0.25 cm^2^ (0.5 cm × 0.5 cm).

**Figure 1:**
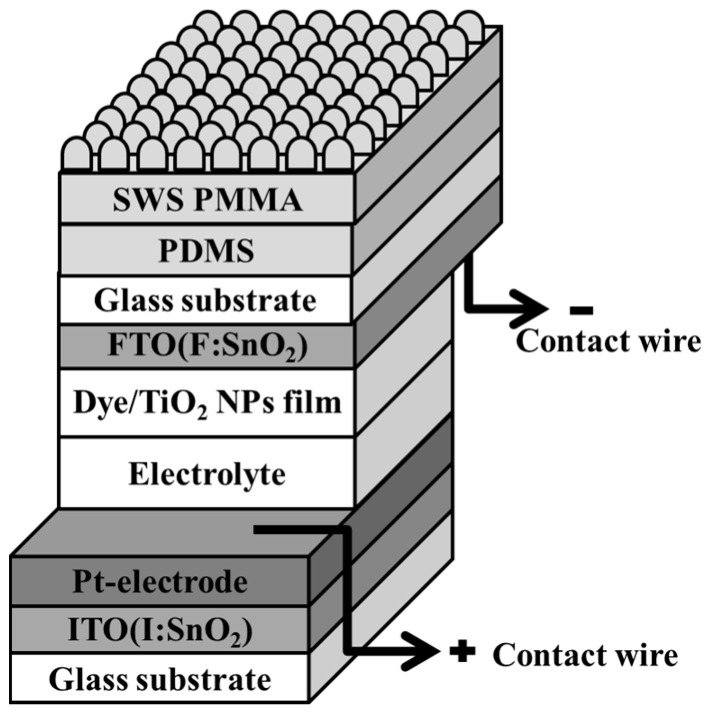
Structure diagram of a subwavelength structured (SWS) anti-reflective film combined with a dye-sensitized solar cell.

Lastly, the photoanode side (where the incident light comes in) of the DSSC device was spin-coated with Polydimethylsiloxane (PDMS) at 300 rpm for 15 s and then at 500 rpm for 20 s. The PDMS solution was made by dissolving PDMS particles into DI water (weight ration of 1:10) with strong agitation. Then the PDMS solution was statically stored in a vacuum chamber for one hour so that the tiny bubbles generated during stirring could be eliminated. After spin-coating the PDMS film, the subwavelength moth-eye structured PMMA film was then attached onto the PDMS layer. The process was completed very carefully, so that no air bubbles were left at the interface. The device was then dried at room temperature for 12 h. Figure 1 shows the structure of the additional moth-eye subwavelength AR layer DSSC device that we have designed and fabricated in this study.

### 2.4. Characterizations and Photoelectrochemical Measurements

The morphologies of PMMA film with a subwavelength moth-eye structure were measured using a field-emission scanning electron microscope (FESEM; JSM-7500F, JEOL, Akishima-shi, Japan). The ultraviolet-visible (UV-Vis) transmittance spectrum of the PMMA film was observed by a UV-Vis spectrophotometer (U-2900, Hitachi High-Technologies Corporation, Tokyo, Japan) with the wavelength ranging from 300 nm to 800 nm at room temperature. Electrochemical impedance spectroscopy (EIS; Zahner Zennium, Kronach, Germany) was performed in the frequency range from 10 mHz to 100 kHz under 1-sun (AM 1.5G) illumination (100 mW/cm^2^), at an applied bias voltage and ac amplitude which were set at the open-circuit voltage of the DSSC and 10 mV, respectively. The incident photon-to-current conversion efficiency (IPCE) was characterized by illuminating the monochromatic light on the devices (with wavelengths from 300 to 800 nm). The light was generated by a 1000 W Xenon arc lamp which was composed of a compact 1/8 meter monochromator (CM110, Spectral Products, Putnam, CT, USA), a color filter wheel (CFW-1-8, Finger Lakes Instrumentation, Lima, NY, USA), and a calibrated photodiode (FDS1010-CAL, Thorlabs Inc., Newton, NJ, USA) with Keithkey 2400 as a source meter (Keithley Instruments, Inc., Cleveland, OH, USA). The current-voltage characteristics of samples were measured by a Keithley 2400 source meter under simulated sunlight (SAN-EI XES-40S1, San Ei Brand, Higashi-Yodogawa, Japan), AM 1.5 radiation at 100 mW/cm^2^.

## 3. Results and Discussion

**Figure 2:**
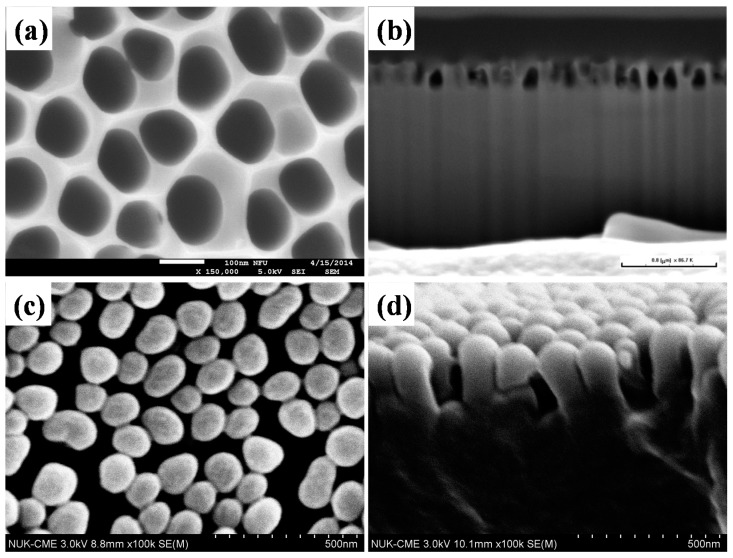
FE-SEM top-view and cross-sectional of an anodic aluminum oxide (AAO) template and polymethyl methacrylate (PMMA) film with subwavelength moth-eye structures. (**a**,**b**) top-view and cross-sectional of AAO template; (**c**,**d**) top-view and cross-sectional of PMMA film with subwavelength moth-eye structures.

Figure 2 shows FESEM images of the surface morphology of an AAO template and subwavelength moth-eye structured PMMA film. The top-view image of the AAO template shown in Figure 2a demonstrates that the pore diameter of the AAO template is around 110 nm. The cross-section image shown in Figure 2b indicates that the pore depth of the AAO template is about 238 nm. Figure 2c shows the top-view image of the PMMA film with subwavelength moth-eye structures made by rolling over the AAO template. The image indicates that the top-end structure of PMMA is the same as the AAO template, with a diameter of around 110 nm. Figure 2d is a cross-sectional image of the PMMA film. The height of the PMMA moth-eye structure is about 234 nm which is also similar to the pore depth of the AAO template. Due to capillary effect, some of the top-end moth-eye structured PMMAs connect to each other with a thin line. Overall, the characteristic tubular pores of the AAO template had been successfully copied to the PMMA film.

**Figure 3:**
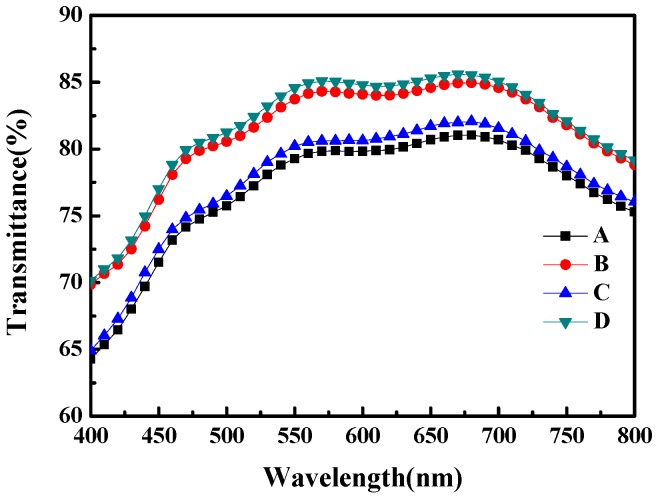
The UV-Vis transmittance spectra. Curves from A to D represent transmittances of bare glass (**sample A**), PDMS/glass (**sample B**), flat PMMA/PDMS/glass (**sample C**) and subwavelength moth-eye structured PMMA/PDMS/glass (**sample D**), respectively.

In order to verify the transmittance of our newly designed AR film, three different structures of AR film were tested. The transmittance of bare glass (sample A) was also characterized for control. The three different AR films tested were PDMS/glass (sample B), flat PMMA/PDMS/glass (sample C) and moth-eye structured PMMA/PDMA/glass (sample D). Figure 3 shows results of transmittance versus wavelength for the four samples. The transmittance data at a wavelength of 550 nm are also listed in Table 1. Coating PDMS on glass would enhance the transmittance since the refractive index of PDMS is 1.42, which is in between glass (1.53) and air (1.00). Thus, comparing sample A with sample B, the transmittance improved 4.47%, increasing from 79.27% to 83.74%. With additional flat PMMA film attached on PDMS/glass (sample C), the transmittance dropped from 83.74% to 80.22% due to the refractive index of PMMA which, being slightly higher than that of PDMS, may cause light reflection at the interface between PDMS and PMMA. With the moth-eye structured PMMA film attached on PDMS/glass (sample D), the transmittance increased up to 84.58% which is the highest value among the four samples with 5.31% improvement, if comparing to that of bare glass. The results indicate that new designed subwavelength moth-eye structured PMMA film indeed enhanced the amount of light that penetrated into the DSSC.

**Figure 4:**
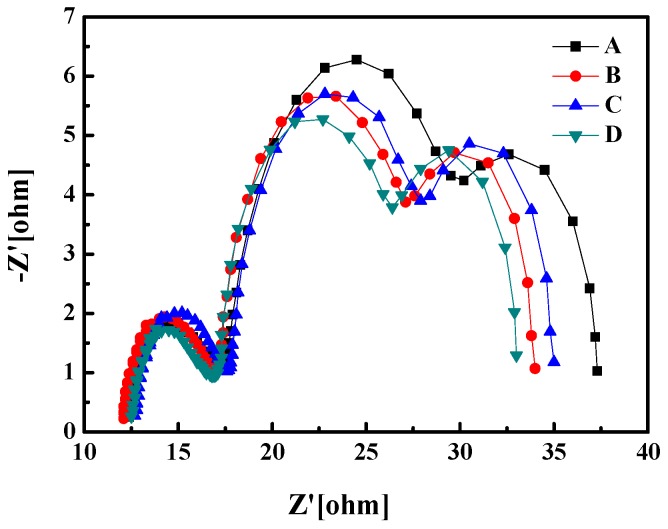
Nyquist plots of the DSSCs. Curves from A to D represent the impedance of bare glass (**sample A**), PDMS/glass (**sample B**), flat PMMA/PDMS/glass (**sample C**) and subwavelength moth-eye structured PMMA/PDMS/glass (**sample D**), respectively.

The four samples were then further assembled to become DSSC devices. Electrochemical impedance spectroscopy was employed to further understand the electron transfer processes in the DSSC. Figure 4 shows the Nyquist plots of samples A to D. Three distinct semicircles are observed in the frequency range of 10 mHz−100 kHz, which are attributed to, from left to right, the electrochemical reaction at the Pt counter electrode and electrolyte interface, the charge transport through the TiO_2_/dye/electrolyte interface, and the Warburg diffusion process of I^−^/I^3−^ in the electrolyte [18,19]. The diameter of the left semicircle refers to the electrical resistance (R_Pt_) at the Pt counter electrode/electrolyte interface. Figure 3 shows that the R_Pt_ of the four samples are similar, indicating that the DSSC fabricating process is stable. The diameter of the middle semicircle corresponds to the recombination resistance (R_rec_) associated with the transport of electrons through the dye/TiO_2_ NPs photoanode/electrolyte interfaces. The right semicircle represents the resistance of I^3−^ ion diffusion in the electrolyte, denoted as R_D_ [20]. The results of the R_rec_ and R_D_ of the four samples are listed in Table 1. The minimum R_rec_ of 9.6 Ω was observed for the DSSC with the subwavelength moth-eye structured PMMA AR film on top of it. This is because the film enhances the penetration of incident light into the dye layer, resulting in the production of more excited electron-and-hole pairs than those without the film. In other words, the film facilitates high carrier density in the DSSC working area. Though the mobility of the carriers may keep the same, the resistivity of specific charge transfer is reduced.

**Figure 5:**
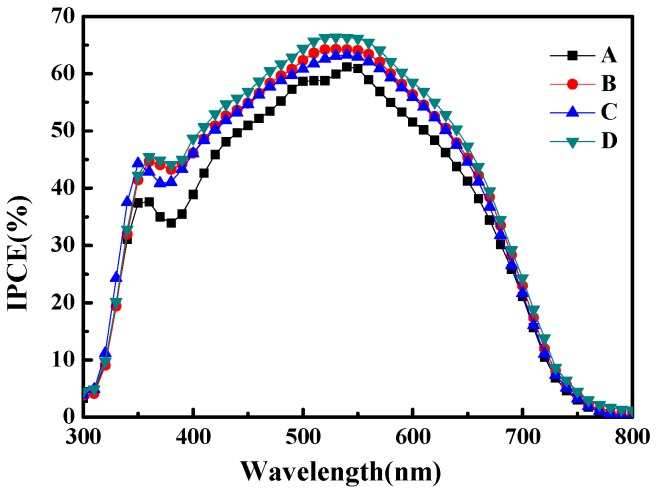
IPC characteristics of the DSSCs. Curves from A to D represent incident monochromatic photon-to-current conversion efficiency of bare glass (**sample A**), PDMS/glass (**sample B**), flat PMMA/PDMS/glass (**sample C**) and subwavelength moth-eye structured PMMA/PDMS/glass (**sample D**), respectively.

Figure 5 shows incident monochromatic photon-to-current conversion efficiency (IPCE) as a function of wavelength for the four samples. The wavelength ranges from 300 to 800 nm. The IPCE represents the number of electron-and-hole pairs that can be generated by monochromatic incident light per unit time. Among the four samples, sample D, which has the moth-eye structured PMMA AR film, shows highest IPCE value of 66.2%. Comparing this value to that of sample A, the IPCE significantly increases 8.7% (from 60.9% to 66.2%). The results imply that sample D has the highest carrier density among the four samples, which is consistent with the minimum R_rec_ observed in the electrochemical impedance spectroscopy.

**Figure 6:**
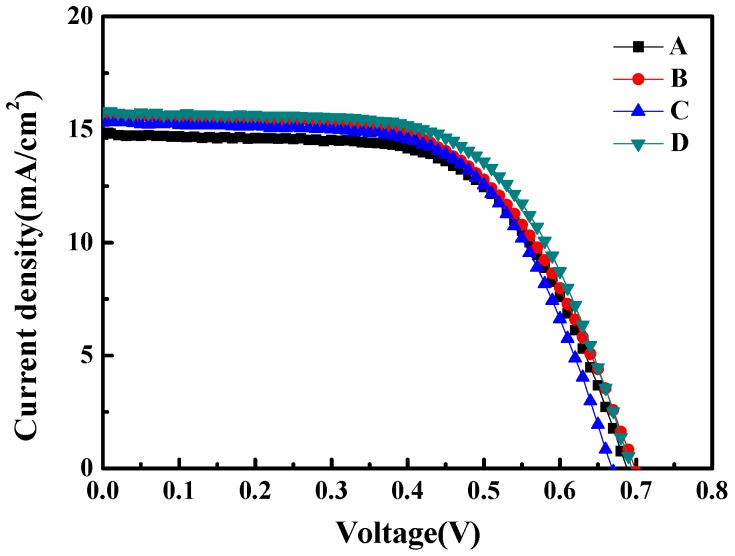
Current density (J) versus voltage (V) characteristics of the DSSCs. Curves from A to D represent the J-V curve of bare glass (**sample A**), PDMS/glass (**sample B**), flat PMMA/PDMS/glass (**sample C**) and subwavelength moth-eye structured PMMA/PDMS/glass (**sample D**), respectively.

Figure 6 shows the current density (J) versus voltage (V) characteristics of samples A to D, measured under AM 1.5G illumination at 100 mW/cm^2^. The open-circuit voltage (VOC) and short-circuit current density (JSC) of the four samples are listed in Table 1. The open-circuit voltage (VOC) is similar among the four samples. The short-circuit current density (JSC), however, increases from 14.77 mA/cm^2^ (sample A) to 15.62 mA/cm^2^ (sample B) with the attachment of the PDMS AR layer. The JSC is further improved to 15.79 mA/cm^2^ (Sample D) by attaching the additional subwavelength moth-eye structured PMMA film on sample B. 

Based on the J-V characteristics, the energy conversion performances of samples A to D were calculated and are also summarized in Table 1. Comparing sample A with sample B, the energy conversion efficiency (η) increases from 6.26% to 6.41% due to the effect of the PDMS AR layer. Comparing sample A with sample D, the energy conversion efficiency (η) increases from 6.26% to 6.79%. Thus, comparing sample B and D, the energy conversion efficiency (η) is improved by 5.93% only by attaching an additional subwavelength moth-eye structured PMMA AR film on the PDMS layer. Sample C, which attached a flat PMMA film on the PDMS layer, has a relatively low energy conversion efficiency of 6.33% because the flat PMMA film could not effectively enhance the light transmittance. Overall, sample D shows the highest energy conversion efficiency of 6.79%.

## 4. Conclusions

In this study, a DSSC attached to a double anti-reflection (AR) layer composed of a subwavelength moth-eye structured PMMA film and a PDMS film was fabricated. The subwavelength moth-eye structured PMMA film was proved to enhance light penetration into the DSSC working area. The light transmittance was enhanced by 5.31% and 4.47% for the case of the double AR layer (subwavelength moth-eye structured PMMA film/PDMS) and the PDMS film alone when compared with bare glass, respectively. The double AR layer DSSC showed the IPCE value of 66.2% at the wavelength of 550 nm, which is 8.7% higher than that of the DSSC without any AR layer. The internal impedance was reduced from 12.9 Ω to 9.6 Ω, the short-circuit current density was increased from 14.77 to 15.79, and the photoelectric conversion efficiency was increased from 6.26% to 6.79% when comparing the DSSC without the AR layer to that with the double AR layer, respectively. More importantly, the process to fabricate the DSSC device with the double AR layer composed of subwavelength moth-eye structured PMMA/PDMS was found to be compatible with the high pressure mechanical compression technology and the high temperature annealing process.

## Figures and Tables

**Table 1:** Characteristics of DSSCs fabricated on the FTO glass.

Sample	Transmittance@550 nm (%)	V_OC_ (V)	J_SC_ (mA/cm^2^)	F.F (%)	η (%)	R_rec_ (Ω)	R_D_ (Ω)
A	79.27	0.69	14.77	61.55	6.26	12.9	7.1
B	83.74	0.70	15.62	58.79	6.41	10.2	6.9
C	80.22	0.67	15.31	61.83	6.33	10.3	7.1
D	84.58	0.69	15.79	61.90	6.79	9.6	6.6
